# Exceptional Values of Meromorphic Function on Annulus

**DOI:** 10.1155/2013/937584

**Published:** 2013-08-05

**Authors:** Hong-Yan Xu

**Affiliations:** Department of Informatics and Engineering, Jingdezhen Ceramic Institute, Jingdezhen, Jiangxi 333403, China

## Abstract

The main purpose of this paper is to study the exceptional values of meromorphic function and its derivative on annulus. We also give some theorems and corollaries about exceptional values of meromorphic function on the annulus, which are the improvement of the previous results given by Chen and Wu.

## 1. Introduction

 It is a very interesting problem on the exceptional values of meromorphic functions in the value distribution theory and argument distributed theory such as the Picard exceptional value and the Borel exceptional value. It is well known that every class of exceptional value is always responding to a singular direction, such as, the Picard exceptional value relating with Julia's direction and the Borel exceptional value relating with Borel's direction. In the 1970s, Gopalakrishna and Bhoosnurmath [1, 2] and Singh and Gopalakrishna [3] investigated Borel exceptional values of meromorphic function and its derivative on the whole complex plane. In 2011, Peng and Sun [4] gave some examples on T exceptional value which is an exceptional value relating with T direction.

In fact, Peng, Sun, Singh, Gopalakrishna, and so forth only studied exceptional values of meromorphic functions in the whole complex plane—single-connected region. For meromorphic function on the double-connected domain and several-connected region, there were few papers about its exceptional values. In 2005, Khrystiyanyn and Kondratyuk [5, 6] proposed the Nevanlinna theory for meromorphic functions on annuli (see also [7]). In 2010, Fernández [8] further investigated the value distribution of meromorphic functions on annuli. In 2012, Xu and Xuan [9] studied the uniqueness of meromorphic functions sharing some values on annuli. At the same year, Chen and Wu [10] firstly studied the Borel exceptional values of meromorphic function and its derivative on annulus. In this paper, we will further investigate the exceptional value of meromorphic function and its derivative on annulus and obtain a series of results which are improvement of previous theorems given by Chen and Wu [10].

The structure of this paper is as follows. In Section 2, we introduce the basic notations and fundamental theorems of meromorphic functions on annulus. Sections 3 and 4 are devoted to study the Borel exceptional values of meromorphic function on annulus and give some consequences of our theorems.  Section 5 are devoted to study the Borel exceptional values of meromorphic function and its derivative on annulus.

## 2. Basic Notions in the Nevanlinna Theory on Annuli

Let *f* be a meromorphic function on the annulus *𝔸* = {*z* : 1/*R*_0_ < |*z* | <*R*_0_}, where 1 < *R* < *R*_0_ ≤ +*∞*. The notations of the Nevanlinna theory on annuli will be introduced as follows. Let

(1) N1(R,f)=∫1/R1n1(t,f)tdt,  N2(R,f)=∫1Rn2(t,f)tdt,m0(R,f)=m(R,f)+m(1R,f),N0(R,f)=N1(R,f)+N2(R,f),

where *n*_1_(*t*, *f*) and *n*_2_(*t*, *f*) are the counting functions of poles of the function *f* in {*z* : *t* < |*z* | ≤1} and {*z* : 1 < |*z* | ≤*t*}, respectively. The Nevanlinna characteristic of *f* on the annulus *𝔸* is defined by

(2) T0(R,f)=m0(R,f)−2m(1,f)+N0(R,f).

Similarly, for $a\in \bar{\mathbb{C}}$, we have

(3) N¯0(r,1f−a)=N¯1(R,1f−a)+N¯2(R,1f−a)=∫1/R1n¯1(t,1/(f−a))tdt +∫1Rn¯2(t,1/(f−a))tdt

in which each zero of the function *f* − *a* is counted only once. In addition, we use ${\bar{n}}_{1}^{k)}(t,1/\left(f-a\right))$ (or ${\bar{n}}_{1}^{(k}(t,1/\left(f-a\right))$) to denote the counting function of poles of the function 1/(*f* − *a*) with multiplicities ≤*k* (or >*k*) in {*z* : *t* < |*z* | ≤1}, each point counted only once. Similarly, we have the notations ${\bar{N}}_{1}^{k)}(t,f)$, ${\bar{N}}_{1}^{(k}(t,f)$, ${\bar{N}}_{2}^{k)}(t,f)$, ${\bar{N}}_{2}^{(k}(t,f)$, and ${\bar{N}}_{0}^{k)}(t,f)$, ${\bar{N}}_{0}^{(k}(t,f)$.

For a nonconstant meromorphic function *f* on the annulus *𝔸* = {*z* : 1/*R*_0_ < |*z* | <*R*_0_}, where 1 < *R* < *R*_0_ ≤ +*∞*, the following properties will be used in this paper (see [5]):

- *T*_0_(*R*, *f*) = *T*_0_(*R*, 1/*f*),
- max⁡{*T*_0_(*R*, *f*_1_ · *f*_2_), *T*_0_(*R*, *f*_1_/*f*_2_), *T*_0_(*R*, *f*_1_ + *f*_2_)} ≤ *T*_0_(*R*, *f*_1_) + *T*_0_(*R*, *f*_2_) + *O*(1),
- *T*_0_(*R*, 1/(*f* − *a*)) = *T*_0_(*R*, *f*) + *O*(1), for every fixed *a* ∈ *ℂ*.

Khrystiyanyn and Kondratyuk [6] also obtained the lemma on the logarithmic derivative on the annulus *𝔸*.

**Theorem 1 (see [6] lemma on the logarithmic derivative)**

Let *f* be a nonconstant meromorphic function on the annulus *𝔸* = {*z* : 1/*R*_0_ < |*z* | <*R*_0_}, where *R*_0_ ≤ +*∞*, and let *λ* > 0. Then

(i) in the case *R*_0_ = +*∞*,
  (4) m0(R,f′f)=O(log⁡(RT0(R,f)))

for *R* ∈ (1, +*∞*) except for the set Δ_*R*_ such that ∫_Δ_*R*__ *R* ^*λ*−1^ *dR* < +*∞*,

(ii) if *R*_0_ < +*∞*, then
  (5) m0(R,f′f)=O(log⁡(T0(R,f)R0−R))

for *R* ∈ (1, *R*_0_) except for the set Δ_*R*_′ such that ∫_Δ_*R*_′_(*dR*/(*R*_0_ − *R*)^*λ*−1^) < +*∞*.

In 2005, The second fundamental theorem on the annulus *𝔸* was first obtained by Khrystiyanyn and Kondratyuk [6]. Cao et al. [11] introduce other forms of the second fundamental theorem on annuli as follows.

**Theorem 2 ([11, Theorem 2.3] The second fundamental theorem)**

Let *f* be a nonconstant meromorphic function on the annulus *𝔸* = {*z* : (1/*R*_0_) < |*z* | <*R*_0_}, where 1 < *R*_0_ ≤ +*∞*. Let *a*_1_, *a*_2_, …, *a*_*q*_ be *q* distinct complex numbers in the extended complex plane $\bar{\mathbb{C}}$. Let *k*_1_, *k*_2_, …, *k*_*q*_ be *q* positive integers, and let *λ* ≥ 0. Then

(6) (q−2)T0(R,f)<∑j=1qN¯0(R,1f−aj)+S(R,f),

where (i) in the case *R*_0_ = +*∞*,

(7) S(R,f)=O(log⁡(RT0(R,f)))

for *R* ∈ (1, +*∞*) except for the set Δ_*R*_ such that ∫_Δ_*R*__ *R* ^*λ*−1^ *dR* < +*∞*,

(ii) if *R*_0_ < +*∞*, then
  (8) S(R,f)=O(log⁡(T0(R,f)R0−R))

for *R* ∈ (1, *R*_0_) except for the set Δ_*R*_′ such that ∫_Δ_*R*_′_(*dR*/(*R*_0_ − *R*)^*λ*−1^) < +*∞*.

**Definition 3**

Let *f*(*z*) be a nonconstant meromorphic function on the annulus *𝔸* = {*z* : 1/*R*_0_ < |*z* | <*R*_0_}, where 1 < *R*_0_ ≤ +*∞*. The function *f* is called a transcendental or admissible meromorphic function on the annulus *𝔸* which provided that

(9) limsup⁡R→∞T0(R,f)log⁡R=∞, 1<R<R0=+∞

or

(10) limsup⁡R→R0T0(R,f)−log⁡(R0−R)=∞, 1<R<R0<+∞,

respectively.

Thus for a transcendental or admissible meromorphic function on the annulus *𝔸*, *S*(*R*, *f*) = *o*(*T*_0_(*R*, *f*)) holds for all 1 < *R* < *R*_0_ except for the set Δ_*R*_ or the set Δ_*R*_′ mentioned in Theorem 1, respectively.

**Definition 4 (see [10], Definition 2.1)**

Let *f*(*z*) be a nonconstant meromorphic function on the annulus *𝔸* = {*z* : 1/*R*_0_ < |*z* | <*R*_0_}, where *R*_0_ = +*∞*. Then the order of *f*(*z*) is defined by

(11) ρ(f)=limsup⁡R→+∞T0(R,f)log⁡R.

**Definition 5 (see [10], Definition 3.1)**

Let *f*(*z*) be a nonconstant meromorphic function of order *ρ*  (0 < *ρ* < *∞*) on the annulus *𝔸* = {*z* : 1/*R*_0_ < |*z* | <*R*_0_}, where 1 < *R*_0_ ≤ +*∞*, and let $a\in \hat{\mathbb{C}}$. Then we say that *a* is

(i) an evB (exceptional value in the sense of Borel) for *f* on *𝔸* for distinct zeros of order ≤*k* if ${\bar{\rho }}_{k}(a,f)<\rho$;
(ii) an evB (exceptional value in the sense of Borel) for *f* on *𝔸* for distinct zeros if $\bar{\rho }(a,f)<\rho$;
(iii) an evB (the Borel exceptional value) for *f* on *𝔸* if *ρ*(*a*, *f*) < *ρ*, where
  (12) ρ¯k(a,f)=limsup⁡R→+∞N¯0k)(R,1/(f−a))log⁡R,ρ¯(a,f)=limsup⁡R→+∞N¯0(R,1/(f−a))log⁡R,ρ(a,f)=limsup⁡R→+∞N0(R,1/(f−a))log⁡R.

In particular, we say that *a* is an evB for *f* on *𝔸* for simple zeros if *k* = 1 and *a* is an evB for *f* on *𝔸* for simple and double zeros if *k* = 2. 

## 3. The Main Results

Now, the main theorems of this paper are listed as follows.

**Theorem 6**

Let *f* be transcendental meromorphic function of order *ρ*  (0 < *ρ* < *∞*) on the annulus *𝔸* : = {*z* : 1/*R*_0_ < |*z* | <*R*_0_}, where *R*_0_ = +*∞*. If there exist $a_{1}^{1},\ldots ,a_{p_{1}}^{1},a_{1}^{2},\ldots ,a_{p_{2}}^{2},\ldots ,a_{1}^{\nu },\ldots ,a_{p_{\nu }}^{\nu }\in \hat{\mathbb{C}}$ such that *a*_1_^*i*^, *a*_2_^*i*^,…, *a*_*p*_*i*__^*i*^ are evBs for *f* on *𝔸* for distinct zeros of order ≤*k*_*i*_, *i* = 1,2,…, *ν*, where *p*_1_,…, *p*_*ν*_, *ν* ∈ *ℕ*_+_, and *k*_1_, *k*_2_,…, *k*_*ν*_ are positive integers or infinity. Then

(13) Ξ:=∑i=1νpiki1+ki+∑i=1ν11+ki∑j=1piδ0ki(aji,f)≤2.

**Remark 7**

Under the assumptions of Theorem 6, if *ν* = 3, *p*_1_ = *r*, *p*_2_ = *t*, *p*_3_ = *s* and *k*_1_ = *k*, *k*_2_ = *l*, *k*_3_ = *m*, since *δ*_0_^*k*_*i*_^(*a*_*j*_^*i*^, *f*) ≥ 0, then we can get

(14) krk+1+tll+1+smm+1≤∑i=1νpiki1+ki +∑i=1ν11+ki∑j=1piδ0ki(aji,f)≤2.

This shows that Theorem 6 is an improvement of results given by Chen and Wu [10].

**Definition 8**

For positive integers *k*, *m*, we define that

(15) δ0k(a,f)=1−limsup⁡R→+∞N0k(R,1/(f−a))T0(R,f),Θ0(a,f)=1−limsup⁡R→+∞N¯0(R,1/(f−a))T0(R,f),

where *N*_0_^*k*^(*R*, 1/(*f* − *a*)) is the counting function of *a*-points of *f* on *𝔸* where an *a*-point of multiplicity *m* is counted *m* times if *m* ≤ *k* and 1 + *k* times if *m* > *k*. In particular, if *k* = *∞*, then

(16) δ0∞(a,f)=δ0(a,f)=liminf⁡R→+∞m0(R,1/(f−a))T0(R,f)=1−limsup⁡R→+∞N0(R,1/(f−a))T0(R,f).

**Theorem 9**

Let *f* be transcendental meromorphic function of order *ρ*  (0 < *ρ* < *∞*) on the annulus *𝔸* : = {*z* : 1/*R*_0_ < |*z* | <*R*_0_}, where *R*_0_ = +*∞*. If there exist $a\in \hat{\mathbb{C}}$ and two positive integers *k* and *p* such that

(17) (1+k)Θ0(a,f)+∑b≠aδ0(b,f)>2−k(p−1),

then there exist at most *p* elements $\hat{\mathbb{C}}\setminus \{a\}$ which are evBs for *f* on *𝔸* for distinct zeros of order not exceeding *k*.

### 3.1. Proof of Theorem 6

**Proof**

For any positive integer *k* or *∞* and $a\in \hat{\mathbb{C}}$, we have

(18) N¯0(R,1f−a)≤k1+kN¯0k)(R,1f−a)+11+kN0k(R,1f−a),

where *k*/(*k* + 1) = 1 and 1/(*k* + 1) = 0 if *k* = *∞*. Then, from (18) and Theorem 2, we have

(19) (∑i=1νpi−2)T0(R,f)  ≤∑i=1νki1+ki∑j=1piN¯0ki)(R,1f−aji)   +∑i=1ν11+ki∑j=1piN0ki(R,1f−aji)+S(R,f).

From Definition 5 and the assumptions of Theorem 6, there exists a constant *η*  (0 < *η* < *ρ*) such that for sufficiently large *R*,

(20) N¯0ki)(R,1f−aji)<Rη, j=1,2,…,pi,i=1,2,…,ν.

Hence, from (19) and (20) and for sufficiently large *R*, we have

(21) (∑i=1νpi−2)T0(R,f)≤∑i=1ν11+ki∑j=1piN0ki(R,1f−aji)+O(Rη)+S(R,f).

Thus, for sufficiently large *R* and taking arbitrary *ε*(>0), we can get from (21) and the definition of *δ*_*k*_(*a*, *f*) that

(22) (∑i=1νpi−2)T0(R,f)  ≤∑i=1ν11+ki∑j=1pi(1−δkiθ(aji,f)+ε)T0(R,f)   +O(Rη)+S(R,f).

Since *R* = +*∞*, it follows that

(23) (Ξ−2−ε)T0(R,f)≤O(Rη)+O(log⁡RT0(R,f)),

for *R* ∈ (1, +*∞*) except for the set Δ_*R*_ such that ∫_Δ_*R*__ *R* ^*λ*−1^ *dR* < +*∞*. If *I* = [*R*, *R*′) ∈ Δ_*R*_, we have

(24) R′λ−Rλ=λ∫RR′xλ−1dx≤λ∫ΔRRλ−1dR<M,

where *M* is a constant. Thus,

(25) (Ξ−2−ε)T0(R,f)≤O(Rη)+O(log⁡RT0(R,f))

holds for all *R*. If *Ξ* > 2, we can choose an arbitrary *ε*  (0 < *ε* < *Ξ* − 2) satisfying *η* + *ε* < *ρ*. Thus, from (25) and for sufficiently large *R*, we can get a contradiction to (13) easily.

Therefore, we can get the conclusion of Theorem 6.

### 3.2. The Proof of Theorem 9

**Lemma 10**

Let *f* be transcendental meromorphic function of order *ρ*  (0 < *ρ* < *∞*) on the annulus *𝔸* : = {*z* : 1/*R*_0_ < |*z* | <*R*_0_}, where 1 < *R*_0_ ≤ +*∞*. Then

(26) limsup⁡R→+∞m0(R,1/f′)T0(R,f)≤2−Θ0(∞,f)−∑b∈ℂδ0(b,f).

**Proof**

Suppose that *b*_1_, *b*_2_,…, *b*_*t*_ ∈ *ℂ* are *t* distinct complex constants. From the definitions of *m*_0_(*R*, *f*), *T*_0_(*R*, *f*), we have

(27) ∑i=1tm0(r,1f−bi)≤m0(r,1f′)+S(R,f)≤T0(R,f′)−N0(R,1f′)+S(R,f).

And since $T_{0}(R,f^{\prime })\leq T_{0}(R,f)+{\bar{N}}_{0}(R,f)+S(R,f)$, then we have

(28) ∑i=1tm0(R,1f−bi)+N0(R,1f′)≤T0(R,f)+N¯0(R,f)+S(R,f).

It follows from the definitions of *δ*_0_(*a*, *f*), Θ_0_(*a*, *f*) that

(29) ∑i=1tδ0(bi,f)+limsup⁡R→+∞N0(R,1/f′)T0(R,f)≤2−Θ0(∞,f).

From (29) and *t* is arbitrary, the proof of Lemma 10 is completed easily.

**Proof of Theorem 9**

Without loss of generality, we assume that *a* = *∞*. Next, we will employ reductio ad absurdum to prove the conclusion of Theorem 9. Suppose that there exist *p* + 1 elements *a*_1_, *a*_2_,…, *a*_*p*+1_ ∈ *ℂ* which are evBs for *f* on *𝔸* for distinct zeros of multiplicity ≤*k*. We know that if *z*_0_ is a zero of *f* − *b* on *𝔸* of multiplicity *s*  (>1) for *b* ∈ *ℂ*, then *z*_0_ is a zero of *f*′ on *𝔸* of multiplicity *s* − 1. It follows that

(30) ∑i=1p+1N¯0(R,1f−ai)≤∑i=1p+1N¯0k)(R,1f−ai)+1kN0(R,1f′).

Therefore, by Theorem 2 we have

(31) pT0(R,f)≤∑i=1p+1N¯0(R,1f−ai)+N¯0(R,f)+S(R,f)≤∑i=1p+1N¯0k)(R,1f−ai)+1kN0(R,1f′) +N¯0(R,f)+S(R,f).

From the definitions of order and evB of *f* on *𝔸*, there exists a constant *η*  (0 < *η* < *ρ*) such that for sufficiently large *R*, we have

(32) N¯0k)(R,1f−ai)<Rη, i=1,2,…,p+1.

From (31), (32), and Lemma 10, for sufficiently large *R*, it follows that

(33) (p−1−1k(2−Θ0(∞,f)−∑b≠∞δ0(b,f))+Θ0(∞,f))   ×T0(R,f)≤Rη+S(R,f),

for *R* ∈ (1, +*∞*) except for the set Δ_*R*_ such that ∫_Δ_*R*__ *R* ^*λ*−1^ *dR* < +*∞*. Since *R*_0_ = +*∞*, similar to the argument as in Theorem 6, from (33) we find that

(34) (p−1−2−(k+1)Θ0(∞,f)−∑b≠∞δ0(b,f)k)T0(R,f)  ≤Rη+O(log⁡(RT0(R,f)))

holds for all *R*. Since *η* < *ρ*, from (34) and for sufficiently large *R*, we can get

(35) (1+k)Θ0(∞,f)+∑b≠∞δ0(b,f)>2−k(p−1),

which is contradiction with the assumption of Theorem 9.

Thus, this completes the proof of Theorem 9.

## 4. Some Consequences of Theorems 6 and 9

 In this section, we will give some consequences of Theorems 6 and 9. Before we give these results, some definitions will be introduced below.

**Definition 11**

Let *f* be meromorphic function on the annulus *𝔸* : = {*z* : 1/*R*_0_ < |*z* | <*R*_0_}, where *R*_0_ = +*∞*. For $a\in \hat{\mathbb{C}}$, then we say that

- *a* is called an exceptional value in the sense of Nevanlinna (evN for short) for *f* on *𝔸*, if *δ*_0_(*a*, *f*) > 0;
- *a* is called a normal value in the sense of Nevanlinna (nvN for short) for *f* on *𝔸*, if *δ*_0_(*a*, *f*) = 0.

In addition, similar to the Picard exceptional value in the whole complex plane, we define that *a* is called an exceptional value in the sense of Picard (evP for short) for *f* on *𝔸*, if *f* has at most a finite number of *a*-points on *𝔸*.

**Consequence 1**

Under the assumptions of Theorem 6, if *k*_1_ = 1, from Theorem 6, we get

(36) p1+2∑i=2νpiki1+ki+∑j=1p1δ01(aj1,f)+∑i=2ν21+ki∑j=1piδ01(aji,f)≤4.

Since *p*_*i*_ ≥ 0 and *δ*_0_^1^(*a*_*j*_^*i*^, *f*) ≥ 0 for *i* = 1,…, *ν*, it follows that

- if *f* has an evB for simple zeros which is also an evN for *f* on *𝔸*, then *f* has at most three evBs for simple zeros on *𝔸*;
- if *a*_1_ and *a*_2_ are two evPs for *f* on *𝔸* then no other element is an evB for *f* for simple zeros on *𝔸*;
- there exist at most four elements which are evBs for *f* simple zeros on *𝔸* since *δ*_1_(*a*_*j*_^1^, *f*) ≥ 0. Moreover, all these four values are nvNs for *f* on *𝔸*.

**Consequence 2**

Under the assumptions of Theorem 6, let *k*_1_ = 1, *p*_1_ = 1, then

(a) if *k*_2_ = 2, we have
  (37) 2p23+∑i=3νpiki1+ki+12δ01(a11,f)+13∑j=1p2δ02(aj2,f)+∑i=3ν11+ki∑j=1piδ0ki(aji,f)≤32.

Since *p*_*i*_ ≥ 0 and *δ*_0_^*k*_*i*_^(*a*_*j*_^*i*^, *f*) ≥ 0, it follows from (37) that *p*_2_ < 3. Thus, if *a*_1_^1^ is an evB for *f* on *𝔸* for simple zeros, that is, *δ*_0_^1^(*a*_1_^1^, *f*) > 0, then there exist at most two other elements which are evBs for *f* on *𝔸* for distinct simple zeros and double zeros. Furthermore,

(i) if *δ*_0_^1^(*a*_1_^1^, *f*) = 1/3, then two other elements evBs are also evNs for *f* on *𝔸*;
(ii) if any of the two other elements *a*_1_^2^ and *a*_2_^2^, say *a*_1_^2^, satisfies *δ*_0_^1^(*a*_1_^2^, *f*) = 1/2, then *a*_1_^1^, *a*_2_^2^ are also evNs for *f* on *𝔸*;
(b) if *k*_2_ = 3, we have
  (38) 3p24+∑i=3νpiki1+ki+12δ01(a1,f)+14∑j=1p2δ03(aj2,f)+∑i=3ν11+ki∑j=1piδ0ki(aji,f)≤32.

From the above inequality and *δ*_0_(*a*, *f*) ≥ 0 for $a\in \hat{\mathbb{C}}$, we get that *p*_1_ = 1, *p*_2_ ≤ 2 and *p*_1_ = 1, *p*_2_ = 2, *p*_*i*_ = 0  (*i* = 3,…, *ν*). Thus, if *f* has an evB for simple zeros on *𝔸*, then there exist at most two other elements which are evBs for *f* distinct zeros of multiplicity ≤3 on *𝔸*. Moreover, all these exceptional values are nvNs for *f* on *𝔸*.

**Consequence 3**

Under the assumptions of Theorem 6, if *k*_1_ = 2, then we have

(39) 2p13+∑i=2νpiki1+ki+13∑j=1p1δ02(aj1,f)+∑i=2ν11+ki∑j=1piδ0ki(aji,f)≤2.

Since ∑_*i*=2_^*ν*^(*p*_*i*_ *k*_*i*_/(1 + *k*_*i*_)) + ∑_*i*=2_^*ν*^(1/(1 + *k*_*i*_))∑_*j*=1_^*p*_*i*_^ *δ*_0_^*k*_*i*_^(*a*_*j*_^*i*^, *f*) ≥ 0, then from (39) we have

(40) 2p13+13∑j=1p1δ02(aj1,f)≤2.

Thus, it follows that *p*_1_ ≤ 3 and *p*_1_ = 3, *p*_*i*_ = 0, *i* = 2,…, *ν*. Hence, we have that *f* has at most three evBs for distinct simple and double zeros on *𝔸*. Moreover, all three evBs for distinct simple and double zeros are nvNs for *f* on *𝔸*.

**Consequence 4**

Under the assumptions of Theorem 6, let *k*_1_ = 2, *p*_1_ = 1.

(a) If *k*_2_ = 1, then we have
  (41) p2+2∑i=3νpiki1+ki+23δ02(a11,f)+∑j=1p2δ01(aj2,f)+∑i=3ν21+ki∑j=1piδ0ki(aji,f)≤83.

Thus, it follows that *p*_2_ ≤ 2. So, if there exists an evB for *f* on *𝔸* for distinct and double zeros, say *a*_1_^1^, then there exist at most two other evBs for *f* on *𝔸* for simple zeros, say *a*_1_^2^, *a*_2_^2^. Furthermore, if *p*_1_ = 1, *p*_2_ = 2, it follows that

(42) 2δ02(a11,f)+3δ01(a12,f)+δ01(a22,f)≤2.

Thus, we can see that any one of *a*_1_^2^, *a*_2_^2^ may not be an evP for *f* on *𝔸*. Furthermore, if *a*_1_^1^ is an evP for *f* on *𝔸*. Then *a*_1_^2^, *a*_2_^2^ are nvNs for *f* on *𝔸*;

(b) if *p*_2_ = 1, *k*_2_ = 3, *k*_3_ = 1, then we have
  (43) p3+∑i=4ν2piki1+ki+23δ02(a11,f)+12δ03(a12,f)+∑j=1p3δ01(aj3,f)+∑i=4ν21+ki∑j=1piδ0ki(aji,f)≤76.

Thus, we can get that *p*_3_ ≤ 1. That is, if *f* have two evBs on *𝔸*, say *a*_1_^1^ and *a*_1_^2^ and *a*_1_^1^ is for distinct simple and double zeros, *a*_1_^2^ for distinct zeros of order ≤3, then *f* has at most one evB for simple zeros on *𝔸*. Furthermore, if *p*_3_ = 1, then

(44) 23δ02(a11,f)+12δ03(a12,f)+δ01(a13,f)≤16.

It follows that *δ*_0_^2^(*a*_1_^1^, *f*) ≤ 1/4, *δ*_0_^3^(*a*_1_^2^, *f*) ≤ 1/3 and *δ*_0_^1^(*a*_1_^3^, *f*) ≤ 1/6. Thus, if any equality of these three inequalities holds, then the other two are nvNs for *f* on *𝔸*.

Now, we will give some consequences of Theorem 9 as follows.

**Consequence 5**

Under the assumptions of Theorem 9, if *k* = 1 and

(45) 2Θ0(a,f)+∑b≠aδ0(b,f)>3−p,

we have the following:

- if *p* = 1 and 2Θ_0_(*a*, *f*) + ∑_*b*≠*a*_ *δ*_0_(*b*, *f*) > 2, then there exists at most one element *b* ≠ *a* which is an evB for *f* on *𝔸* for simple zeros; in particular, this holds if there exists an $a\in \hat{\mathbb{C}}$ satisfying Θ_0_(*a*, *f*) = 1;
- if *p* = 2 and 2Θ_0_(*a*, *f*) + ∑_*b*≠*a*_ *δ*_0_(*b*, *f*) > 1, then there exists at most two elements *b*_1_, *b*_2_ ≠ *a* which are evBs for *f* on *𝔸* for simple zeros; in particular, this holds if there exists an $a\in \hat{\mathbb{C}}$ satisfying Θ_0_(*a*, *f*) > 1/2;
- if *p* = 3 and 2Θ_0_(*a*, *f*) + ∑_*b*≠*a*_ *δ*_0_(*b*, *f*) > 0, then there exists at most three elements *b*_1_, *b*_2_, *b*_3_ ≠ *a* which are evBs for *f* on *𝔸* for simple zeros; in particular, this holds if there exists an $a\in \hat{\mathbb{C}}$ satisfying Θ_0_(*a*, *f*) > 0.

**Remark 12**

Under the assumptions of Theorem 9, from Consequence 5, we have that if there exist four distinct elements $b_{1},b_{2},b_{3},b_{4}\in \hat{\mathbb{C}}$ which are evBs for *f* on *𝔸* for simple zeros, then Θ_0_(*b*_*i*_, *f*) ≤ 1/2 and Θ_0_(*a*, *f*) = 0 for *a* ≠ *b*_*i*_ and *i* = 1,2, 3,4.

**Consequence 6**

Under the assumptions of Theorem 9, if *k* = 2 and

(46) 3Θ0(a,f)+∑b≠aδ0(b,f)>4−2p,

we have the following:

- if *p* = 1 and 3Θ_0_(*a*, *f*) + ∑_*b*≠*a*_ *δ*_0_(*b*, *f*) > 2, then there exists at most one element *b* ≠ *a* which is an evB for *f* on *𝔸* for distinct simple and double zeros; in particular, this holds if there exists an $a\in \hat{\mathbb{C}}$ satisfying Θ_0_(*a*, *f*) > 2/3;
- if *p* = 2 and 3Θ_0_(*a*, *f*) + ∑_*b*≠*a*_ *δ*_0_(*b*, *f*) > 0, then there exists at most two elements *b*_1_, *b*_2_ ≠ *a* which are evBs for *f* on *𝔸* for distinct simple and double zeros; in particular, this holds if there exists an $a\in \hat{\mathbb{C}}$ satisfying Θ_0_(*a*, *f*) > 0.

**Remark 13**

Under the assumptions of Theorem 9, from Consequence 6, we have that if there exist four distinct elements $b_{1},b_{2},b_{3}\in \hat{\mathbb{C}}$ which are evBs for *f* on *𝔸* for distinct simple and double zeros, then Θ_0_(*b*_*i*_, *f*) ≤ 2/3 and Θ_0_(*a*, *f*) = 0 for *a* ≠ *b*_*i*_ and *i* = 1,2, 3.

## 5. Exceptional Value of Meromorphic Function and Its Derivative

 In this section, we will study the exceptional value of meromorphic function *f* on *𝔸* and its differential polynomial of the form

(47) M[f]:=M[f,f′,…,f(k)]=af(z)i0(f′)i1⋯(f(k))ik,

where *i*_0_, *i*_1_,…, *i*_*k*_ ∈ *ℕ*, *k* ∈ *ℕ*_+_, and *a*(≠0) ∈ *ℂ* is a complex constant.

**Theorem 14**

Let *f* be transcendental meromorphic function of order *ρ*  (0 < *ρ* < *∞*) on the annulus *𝔸* : = {*z* : 1/*R*_0_ < |*z* | <*R*_0_}, where *R*_0_ = +*∞*. If 0 and *∞* are evBs for *f* on *𝔸* for distinct zeros, then *M*[*f*] has no evB for simple zeros in $\hat{\mathbb{C}}\setminus \{0,\infty \}$.

**Proof**

From (21), set *σ* = *i*_0_ + *i*_1_ + ⋯+*i*_*k*_. Since *f* is a transcendental meromorphic function on *𝔸*, similar to the method of [12, Theorem 1.21], we can get that *f* and *M*[*f*] have the same order *ρ* on *𝔸*. By applying Theorem 1 for *M*[*f*], for *b*(≠0) ∈ *ℂ* we have

(48) T0(R,M[f])≤N¯0(R,1M[f])+N¯0(R,M[f]) +N¯0(R,1M[f]−b)+S(R,M[f])≤N¯0(R,1M[f])+N¯0(R,f) +12N¯01)(R,1M[f]−b) +12N¯01(R,1M[f]−b)+S(R,f)≤N¯0(R,1M[f])+N¯0(R,f) +12N¯01)(R,1M[f]−b) +12T0(R,f)+S(R,f).

Since

(49) N¯0(R,1M[f])≤N0(R,fσM[f])+N¯0(R,1fσ)≤T0(R,M[f]fσ)+N¯0(R,1f)+S(R,f)≤N0(R,M[f]fσ)+N¯0(R,1f)+S(R,f)≤O(N¯0(R,1f)+N¯0(R,f))+S(R,f).

and 0 and *∞* are evBs for *f* on *𝔸* for distinct zeros, then there exists a constant *η*  (0 < *η* < *ρ*) such that for sufficiently large *R*, we have

(50) N¯0(R,1f)<Rη,  N¯0(R,f)<Rη.

Suppose that *b* is an evB for *M*[*f*] on *𝔸* for simple zeros, since *M*[*f*] is of order *ρ*. Then for sufficiently large *R*, there exists a constant *β* < *ρ* such that

(51) N¯01)(R,1M[f]−b)<Rβ.

Thus, from (48)–(50), it follows that

(52) 12T0(R,M[f])≤O(rβ′)+O(log⁡RT0(R,f)),

where *β*′ = max⁡{*η*, *β*} and for *R* ∈ (1, +*∞*) except for the set Δ_*R*_ such that ∫_Δ_*R*__ *R* ^*λ*−1^ *dR* < +*∞*. Since *M*[*f*] is of order *ρ*, then there exists a sequence {*R*_*n*_} tending to *∞* such that

(53) T0(Rn,M[f])>Rnα,

where *α*  (*β*′ < *α* < *ρ*) is a constant. Thus, we can get a contradiction by using the same argument as in Theorem 6.

Therefore, *M*[*f*] has no evB for simple zeros in $\hat{\mathbb{C}}\setminus \{0,\infty \}$.
